# A comprehensive learning based swarm optimization approach for feature selection in gene expression data

**DOI:** 10.1016/j.heliyon.2024.e37165

**Published:** 2024-09-02

**Authors:** Subha Easwaran, Jothi Prakash Venugopal, Arul Antran Vijay Subramanian, Gopikrishnan Sundaram, Beebi Naseeba

**Affiliations:** a Department of Science and Humanities, Karpagam College of Engineering, Myleripalayam Village, Coimbatore-641032, Tamilnadu, India; b Department of Information Technology, Karpagam College of Engineering, Myleripalayam Village, Coimbatore-641032, Tamilnadu, India; c Department of Computer Science and Engineering, Karpagam College of Engineering, Myleripalayam Village, Coimbatore-641032, Tamilnadu, India; d School of Computer Science and Engineering, VIT-AP University, Amaravathi-522241, Andhra Pradesh, India

**Keywords:** Comprehensive learning, Feature selection, Gene expression, Gene selection, Swarm intelligence, Cancer classification

## Abstract

Gene expression data analysis is challenging due to the high dimensionality and complexity of the data. Feature selection, which identifies relevant genes, is a common preprocessing step. We propose a Comprehensive Learning-Based Swarm Optimization (CLBSO) approach for feature selection in gene expression data. CLBSO leverages the strengths of ants and grasshoppers to efficiently explore the high-dimensional search space. Ants perform local search and leave pheromone trails to guide the swarm, while grasshoppers use their ability to jump long distances to explore new regions and avoid local optima. The proposed approach was evaluated on several publicly available gene expression datasets and compared with state-of-the-art feature selection methods. CLBSO achieved an average accuracy improvement of 15% over the original high-dimensional data and outperformed other feature selection methods by up to 10%. For instance, in the Pancreatic cancer dataset, CLBSO achieved 97.2% accuracy, significantly higher than XGBoost-MOGA's 84.0%. Convergence analysis showed CLBSO required fewer iterations to reach optimal solutions. Statistical analysis confirmed significant performance improvements, and stability analysis demonstrated consistent gene subset selection across different runs. These findings highlight the robustness and efficacy of CLBSO in handling complex gene expression datasets, making it a valuable tool for enhancing classification tasks in bioinformatics.

## Highlights

• Introduced CLBSO for efficient feature selection in gene expression analysis.
• CLBSO outperforms traditional swarm algorithms in classifier efficacy.
• Validated stability of CLBSO with Jaccard Index across cancer datasets.
• Demonstrated CLBSO's quick convergence to optimal solutions.

## 1 Introduction

The progression of microarray technologies has been notably swift in the era following the completion of the human genome project, allowing for simultaneous analysis of the expression levels of numerous genes. This advancement, while significant, introduces complexities due to the inherently high-dimensional nature of gene expression data, combined with typically small sample sizes used in experiments [1]. Addressing these complexities is crucial for effective biomarker discovery, accurate cancer diagnosis, and the precise differentiation of tumor types, which are central challenges in the post-analysis stages of microarray studies. The implementation of an advanced feature selection mechanism is essential in mitigating these challenges by simplifying the data's dimensionality [2].

Feature selection in the context of microarray analysis, also known as gene or variable reduction, aims to identify a critical subset of informative features. This is achieved by removing irrelevant or redundant data from the initial set, thereby focusing on features of utmost relevance. Such a process is instrumental in uncovering potential biomarkers for diseases and facilitating the construction of effective disease classifiers, particularly in the realm of oncology [3]. There exists a plethora of feature selection methods, broadly categorized into filters, wrappers, embedded, and hybrid approaches. Filters evaluate features based on various metrics like distance, information theory, consistency, and dependency without depending on any classifiers [4], [5], [6]. Wrapper methods, on the other hand, select features based on the predictive accuracy of specific classifier models, often achieving better results than filter methods, albeit at the expense of computational efficiency. Embedded methods integrate feature selection as part of the model training process, representing a hybrid of the previous two. Finally, hybrid methods combine the initial screening power of filter methods with the model-specific optimizations of wrapper methods [7].

The outcomes of feature selection are generally twofold: the ranking of features according to their importance or the selection of a subset of features. While ranking methods list features by their significance, subset selection methods provide a definitive group of features for further analysis. The stability of feature selection processes is pivotal, especially when dealing with high-dimensional data like that from microarray studies. This stability refers to the ability of the feature selection process to produce consistent results under different data conditions, which is essential for the reliable identification of genetic markers and the development of accurate disease classifiers [8]. Efforts to improve feature selection stability have led to the development of methods focusing on sample weighting, group-based selection, and ensemble approaches [9].

The Comprehensive Learning-Based Swarm Optimization (CLBSO) method introduced in this study represents a novel approach to feature selection in gene expression analysis. Unlike the group feature selection and ensemble methods, the CLBSO leverages the natural foraging behaviors of ants and grasshoppers to navigate the complex, high-dimensional search space effectively, adapting to data variability without the need for pre-clustering or algorithm amalgamation.

This research makes several significant contributions:

• The proposition and elaboration of the CLBSO algorithm, inspired by the foraging patterns of ants and grasshoppers, for the effective balance between local and global search capabilities, improving the feature selection process in gene expression datasets.
• The introduction of a novel adaptive weighting strategy, enhancing the algorithm's ability to modify the impact of local and global search phases dynamically, which in turn aids in more accurately pinpointing relevant features for disease classification.
• The comprehensive testing of the CLBSO algorithm across various cancer gene expression profiles, displaying its superior performance in metrics such as accuracy and F-measure against other leading optimization techniques like XGBoost-MOGA, ISSA, BCOOT, and SBCSO.
• An in-depth analysis of the CLBSO's performance, highlighting its consistency in identifying compact, significant feature sets across different types of cancer, thereby aiding in a deeper understanding of cancer mechanisms and aiding in the development of targeted treatments.
• The demonstration of the versatility of the CLBSO algorithm, illustrating its potential application beyond the realm of gene expression to other feature selection scenarios in diverse fields.

The remainder of this paper is structured as follows: Section 2 reviews relevant literature; Section 3 details the CLBSO methodology; Section 4 describes the implementation; Section 5 presents experimental results; Section 6 concludes the study.

## 2 Related works

Current studies underline the significance of implementing advanced learning mechanisms for in-depth analysis of gene characteristics, which subsequently enhance classification efficacy [10]. The application of evolutionary strategies like Particle Swarm Optimization (PSO), Artificial Bee Colony (ABC), and Genetic Algorithms (GA) has been prominent in the gene selection arena. Additionally, methodologies incorporating Artificial Neural Networks (ANNs), Fuzzy Logic Systems (FLS), and the Hybrid Stem Cell (HSC) algorithm have demonstrated efficacy in classification tasks [11], [12], [13], [14], [15]. Particularly, GA has garnered recognition for its efficiency in sifting through a myriad of potential solutions to identify the most appropriate gene subsets. Furthermore, the realm of gene selection has witnessed the ascendancy of swarm optimization strategies, offering a robust mechanism for dimensionality reduction through the principles of swarm intelligence, culminating in optimal solutions [16], [17], [18], [19].

The challenge posed by a substantial feature space, typically laden with irrelevant or redundant genes, necessitates the adoption of gene selection for enhanced classification outcomes in both machine learning and medical sciences. Recent trends point towards the integration of hybrid machine learning techniques, particularly metaheuristic optimization, for the discernment of pertinent and informative genes. Innovations such as a hybrid multi-objective cuckoo search complemented by evolutionary operators have demonstrated superiority in gene selection, particularly across high-dimensional cancer microarray datasets [20], [21], [22], [23]. Likewise, hybridized harmony search optimization approaches have shown promise in addressing feature selection within high-dimensional data classification, surpassing other established algorithms in efficiency [24], [25], [26].

The domain of swarm intelligence optimization algorithms has been acknowledged for its substantial contribution to feature selection, attributed to their extensive global search capabilities and inherent simplicity. Notable developments include the integration of teaching-learning-based optimization (TLBO) and gravitational search algorithms (GSA) into a cohesive hybrid wrapper algorithm [27]. This innovative approach combines mRMR for initial gene relevance determination with a subsequent selection of informative genes through a refined method [28]. Further advancements have been made with the introduction of the multidimensional population-based bacterial colony optimization (BCO-MDP) for classification-oriented feature selection [29], alongside methodologies leveraging the gray wolf optimization algorithm and the Harris Hawk optimization algorithm, each tailored for nuanced feature selection in gene expression analysis. Recent explorations into feature selection have also incorporated improved moth-flame optimization algorithms and the synergistic use of ant colony optimization with RelieF for enhanced tumor classification [30], [31].

Additionally, there have been significant advancements in machine learning techniques for disease prediction and classification, as seen in recent studies focusing on DNA sequence classification [32], [33]. These studies highlight the growing role of machine learning and deep learning in enhancing diagnostic accuracy and treatment outcomes across various medical applications.

### 2.1 Research gap

Despite the extensive array of feature selection methodologies documented in the literature, a discernible gap persists, particularly regarding a method that seamlessly combines efficiency and effectiveness for gene expression data analysis. Common challenges encountered by existing techniques include prohibitive computational demands, susceptibility to overfitting, and difficulties in navigating high-dimensional datasets. Moreover, there is a notable deficiency in the integration of varied optimization methods to harness their combined strengths for a more comprehensive exploration of the search space.

Addressing these concerns, the proposed Comprehensive Learning-Based Swarm Optimization (CLBSO) approach innovatively employs ant and grasshopper behaviors for an advanced navigation of the complex, high-dimensional search landscape, adjusting adeptly to data changes. This method uniquely combines ants for meticulous local searches with grasshoppers for expansive global searches, ensuring a thorough exploration and avoidance of local optimum pitfalls. The approach further incorporates an educational strategy, enhancing the swarm's adaptability to data shifts, thereby refining the selection process's overall effectiveness. The CLBSO methodology aims to bridge the current research void by presenting a balanced, efficient, and effective solution for gene expression data analysis, capitalizing on the synergistic potential of ants and grasshoppers to systematically explore and adapt to the evolving search landscape.

## 3 The proposed methodology

### 3.1 Problem definition

Given a gene expression dataset *D* consisting of *n* samples and *m* features (genes), the objective is to find a subset of features S⊆{1,2,…,m} that maximizes a specific criterion, such as classification accuracy, while minimizing the number of selected features. Formally, the problem can be defined as shown in equation (1) below:

(1) $$\max_{S\subseteq 1,2,\ldots ,m}f(S)$$

where f(S) is a fitness function that measures the quality of the selected feature subset *S*. The fitness function aims to maximize the classification accuracy.

### 3.2 Proposed CLBSO algorithm

**Figure 1:**
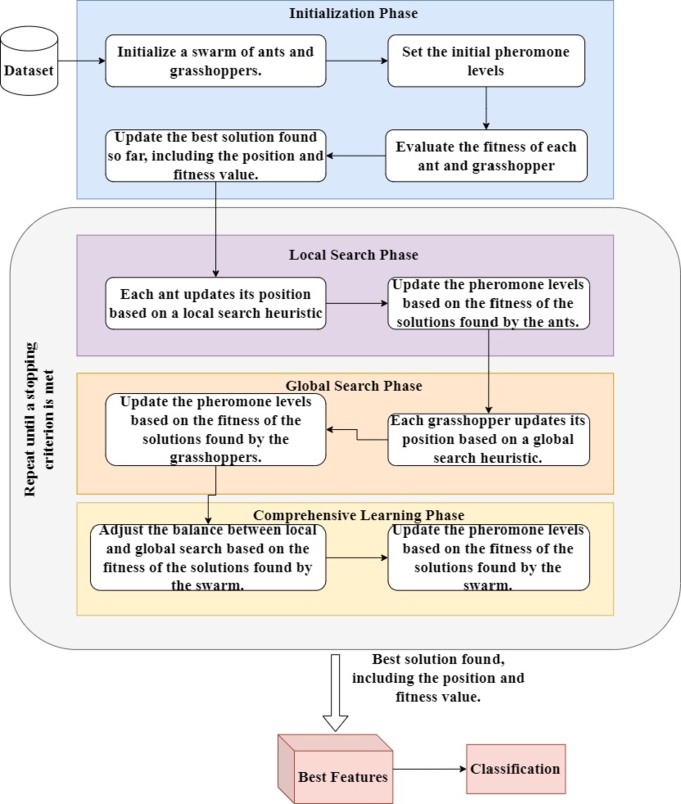
Architecture of the proposed Comprehensive Learning-Based Swarm Optimization (CLBSO) model. The diagram details the Initialization Phase, Local Search Phase, Global Search Phase, and Comprehensive Learning Phase, illustrating the flow and interactions within the algorithm.

**Figure 2:**
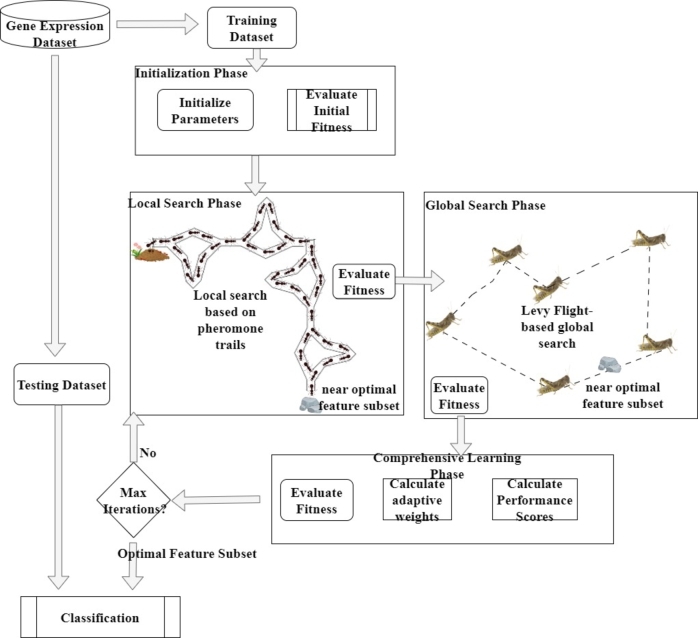
Working of the proposed CLBSO Feature Selection Approach.

The proposed Comprehensive Learning-Based Swarm Optimization (CLBSO) method leverages swarm intelligence principles to tackle optimization challenges characterized by intricate and dynamic environments. The architecture of the proposed CLBSO is shown in Fig. 1. It integrates the distinct yet synergistic strategies of ants and grasshoppers to create a robust optimization framework capable of adapting to changes within its operational landscape. The CLBSO algorithm unfolds through four key stages: 1) Initialization Phase, 2) Local Search Phase, 3) Global Search Phase, and 4) Adaptation or Comprehensive Learning Phase, as depicted in Fig. 2. The following subsections elucidate each stage within the CLBSO algorithm's workflow.

In contrast to traditional approaches, the CLBSO leverages a singular swarm population that incorporates the searching mechanisms of both ants and grasshoppers. This innovative structure allows each potential solution within the swarm to exhibit characteristics of both an “ant” and a “grasshopper,” thereby enhancing the diversity and adaptability of the search process. The synergy between ant-like and grasshopper-like behaviors within the CLBSO framework is encapsulated in the unified optimization strategy discussed in the following sections.

#### 3.2.1 Initialization phase

Metaheuristic feature selection algorithms typically employ binary encoding to represent the solution space effectively, facilitating both the representation of feature subsets and the simplification of algorithmic complexity. This binary approach assigns a binary vector of length n to each solution, where n denotes the total number of available features. In this vector, each bit corresponds to a specific feature: a “1” indicates the inclusion of the feature in the selected subset, while a “0” denotes its exclusion.

In this research, we adhere to this binary representation strategy to maximize the inherent advantages of the algorithm. During the Initialization Phase, the CLBSO algorithm generates an initial population of solutions, represented as binary strings, which correspond to various feature subset configurations within the gene expression data context. This initial generation process is randomized to ensure a diverse starting point for the optimization journey. Each individual's binary string reflects its proposed feature subset, and the corresponding objective function values are computed to assess the quality and efficacy of each proposed solution.

Let *N* represent the number of individuals in the population, and let *n* denote the number of features in the gene expression dataset. The positions of individuals are represented by binary strings $X_{i}$, where i=1,2,…,n. The initialization process can be described as follows:

(a) Generate random positions for individuals as per equation (2):

(2) $$X_{i}=\left\{ \begin{array}{cc} 1, & \text{with probability}\;p\\ 0, & \text{with probability}\;1-p\; \end{array}\right.$$

where *p* is the initial probability of selecting a feature.

(b) Calculate the objective function value f(X) shown in equation (3) for each individual. A commonly used objective function for feature selection problems is a combination of classification performance (e.g., accuracy) and a penalty term that encourages smaller feature subsets. One such objective function can be defined as follows:

(3) $$f(X)=w_{1}\cdot \text{accuracy}(X)-w_{2}\cdot \frac{\text{num}\_\text{selected}\_\text{features}(X)}{n}$$

where *X* represents an individual in the population (i.e., a binary string encoding a feature subset), accuracy(X) is the classification accuracy of a chosen classifier trained on the selected features, the num_selected_features(X) is the number of selected features in the subset, *n* is the total number of features in the gene expression dataset, and $w_{1}$ and $w_{2}$ are weighting factors that balance the importance of classification performance and feature subset size. The classifier used is SVM.

During the Initialization Phase, the objective function value f(X) is calculated for each individual in the population. This value will be used to guide the search process in the Local Search Phase, Global Search Phase, and Adaptation Phase.

#### 3.2.2 Local search phase

During the Local Search Phase within the CLBSO framework, the algorithm employs the ‘ant’ mechanism to conduct an intensive search within the local vicinity of the existing population. This stage is crucial for thoroughly exploring promising areas within the search space, enabling the algorithm to refine and enhance potential solutions.

(a) Pheromone Matrix Update: Central to this phase is the concept of a pheromone matrix, which symbolizes the features' appeal to the ants. Post each cycle, this matrix's values are adjusted reflecting the solution qualities encountered. For any given feature *j*, the pheromone level adjustment is articulated as shown in equation (4):

(4) $$\tau_{j}=(1-\rho )\cdot \tau_{j}+\Delta \tau_{j}$$

where *ρ* symbolizes the rate of pheromone fading, existing in the interval (0, 1), and $\Delta \tau_{j}$ signifies the pheromone increment, directly tied to the performance of solutions incorporating feature *j*.

(b) Ant-led Local Modification: Within this step, each entity $X_{i}$ within the swarm undergoes a transformation by an ant, which probabilistically toggles features guided by the prevailing pheromone levels. The likelihood of a feature *j* being toggled for the entity $X_{i}$ is delineated as shown in equation (5):

(5) $$p_{ij}=\frac{\tau_{j}^{\alpha }\cdot {\left(1-\tau_{j}\right)}^{\beta }}{\sum_{k=1}^{n}\tau_{k}^{\alpha }\cdot {\left(1-\tau_{k}\right)}^{\beta }}$$

Here, *α* and *β* serve to balance the influence of pheromone concentration against its inverse, shaping the decision-making process.

(c) Solution Refinement: Subsequent to the ants' local search endeavors, the algorithm updates the solutions. An enhanced solution, demonstrating superior objective function performance over its predecessor, supplants the latter.

(d) Objective Function Reevaluation: The phase concludes with a recalibration of the objective function values for the newly adjusted solutions, in accordance with the criteria established during the Initialization Phase.

By fostering detailed exploration within close proximity of the extant solutions, the Local Search Phase ensures the algorithm's proficiency in exploiting accessible regions within the search space. Following the completion of this phase, the algorithm transitions into the Global Search Phase. Here, the ‘grasshopper’ components engage in Levy flights, aiming to explore broader, uncharted territories of the search domain.

#### 3.2.3 Global search phase

The Global Search Phase in the CLBSO framework is characterized by the ‘grasshopper’ elements employing Levy flights for extensive search activities. This phase is designed to propel the algorithm beyond local optima by venturing into unexplored territories of the search space through significant, randomized leaps, drawing from the Levy distribution principle.

(a) Implementation of Levy Flight: Each grasshopper, represented as $X_{i}$ within the swarm, is relocated according to the equation (6) shown below:

(6) $$X_{i}^{\prime }=X_{i}+s\cdot L(s)$$

Here, *s* denotes a positive step size, and L(s) signifies a function governed by the Levy distribution, characterized by its probability density function shown in equation (7) below:

(7) $$p(L)=\frac{\exp(-\frac{1}{2}{\left(\frac{\sigma }{L}\right)}^{2})}{\sqrt{2\pi }L^{3}}$$

In this context, *σ* acts as a scale factor that modulates the distribution's spread.

(b) Conversion to Binary Format: Given the binary nature of our search domain, it is imperative to transform $X_{i}^{\prime }$ into a binary format. This transition is achieved through equation (8) as follows:

(8) $$X_{ij}^{\prime }=\left\{ \begin{array}{cc} 1, & \text{if}\;X_{ij}^{\prime }>T\\ 0, & \text{otherwise} \end{array}\right.$$

where $X_{ij}^{\prime }$ denotes the *j*-th component of the new position $X_{i}^{\prime }$, with *T* serving as the demarcation threshold.

(c) Solution Enhancement: The algorithm adopts the new position $X_{i}^{\prime }$ over the old $X_{i}$ if the former exhibits superior performance based on the objective function assessment.

(d) Recalculation of Objective Function: Following the update, a fresh computation of the objective function values is undertaken for the newly adjusted solutions, aligning with the parameters set forth in the Initialization Phase.

By facilitating exploration across broader and potentially more promising areas of the search space, the Global Search Phase crucially prevents the algorithm from succumbing to local optimum traps. Subsequent to this phase, the CLBSO algorithm transitions into the Adaptation or Comprehensive Learning Phase, which tailors the search mechanism adaptively, informed by accumulated historical insights.

#### 3.2.4 Comprehensive learning phase

The Comprehensive Learning Phase in the CLBSO framework is where the algorithm dynamically refines its exploration and exploitation strategies by integrating the methodologies of both ants and grasshoppers, contingent upon their respective successes in the current search context. The performance of each group is assessed based on their individual contributions towards discovering the optimal solution to date.

(a) Performance Evaluation of Ants and Grasshoppers: For each feature *j* within the search domain, the effectiveness of ants ($S_{A_{j}}$) and grasshoppers ($S_{G_{j}}$) is determined through equations (9) and (10) shown below:

(9) $$S_{A_{j}}=\sum_{i=1}^{N}\frac{f_{A}(X_{i})}{F_{\text{max}}}\cdot X_{ij}$$

(10) $$S_{G_{j}}=\sum_{i=1}^{N}\frac{f_{G}(X_{i})}{F_{\text{max}}}\cdot X_{ij}$$

Here, *N* denotes the total solution count within the population, $f_{A}(X_{i})$ and $f_{G}(X_{i})$ represent the objective function scores attributed to ants and grasshoppers within the context of solution $X_{i}$, respectively, and $F_{\text{max}}$ is the peak objective function value identified across all present solutions.

(b) Adaptive Weight Calculation: The adaptive weights for ants ($W_{A}$) and grasshoppers ($W_{G}$) are derived from their respective performance metrics as shown in equations (11) and (12):

(11) $$W_{A}=\frac{\sum_{j=1}^{D}S_{A_{j}}}{\sum_{j=1}^{D}(S_{A_{j}}+S_{G_{j}})}$$

(12) $$W_{G}=\frac{\sum_{j=1}^{D}S_{G_{j}}}{\sum_{j=1}^{D}(S_{A_{j}}+S_{G_{j}})}$$

with *D* representing the dimensionality of the feature space.

(c) Solution Adaptation via Adaptive Weights: Solutions are then adjusted reflecting the synergized influence of ants' and grasshoppers' search mechanisms, as modulated by the calculated adaptive weights using equation (13):

(13) $$X_{ij}^{t+1}=X_{ij}^{t}+W_{A}\cdot A_{j}+W_{G}\cdot G_{j}$$

where $X_{ij}^{t+1}$ is the newly adjusted value for feature *j* in solution $X_{i}$ for the next iteration t+1, with $A_{j}$ and $G_{j}$ denoting the respective updates from ants and grasshoppers for that feature.

(d) Objective Function Recalculation: Subsequent to the adjustments, a reevaluation of the objective function values is conducted for the newly updated solutions, adhering to the foundational metrics established in the Initialization Phase.

By amalgamating the distinct capabilities of ants for meticulous local scrutiny and grasshoppers for expansive global ventures, the Comprehensive Learning Phase equips the CLBSO algorithm with a refined mechanism for navigating the search space. This innovative approach significantly augments the algorithm's capacity for a more harmonized exploration and exploitation, thereby enhancing its overall efficacy in high-dimensional space analysis. The pseudocode of the proposed CLBSO is shown in Algorithm 1.

**Algorithm 1:**
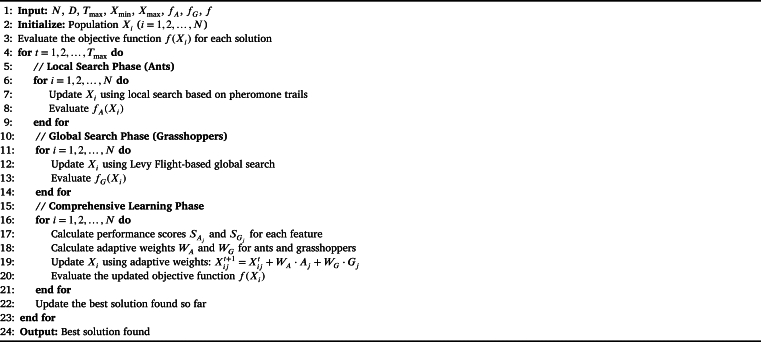
Comprehensive Learning-Based Swarm Optimization (CLBSO).

## 4 Experimental setup and evaluation

All experiments were conducted using the existing high-performance hardware available in our lab. We utilized a system equipped with an NVIDIA A100 GPU, an Intel i9 processor, 64 GB of RAM, and 1TB of SSD storage, which provided the necessary computational power and efficiency for handling large datasets and complex computations. The software environment was built on Ubuntu 18.04. This setup ensured efficient processing and accurate results, highlighting the advantage of having access to advanced computational resources. The NVIDIA A100 GPU significantly accelerated deep learning tasks, while the Intel i9 processor and 64 GB RAM facilitated smooth execution of various computational processes. The fast SSD storage supported efficient data handling, essential for deep learning experiments. These resources were crucial for achieving the high levels of performance required for the study.

### 4.1 Datasets

The proposed CLBSO algorithm is evaluated using seven benchmark cancer datasets sourced from the Curated Microarray Database (CuMiDa) repository [34]. The datasets encompass diverse cancer types and exhibit varying sample and gene quantities, thereby providing a comprehensive evaluation of the algorithm's feature selection efficacy. Table 1 displays the specifics of the datasets. The Pancreatic dataset (GSE16515) contains 54,676 genes across 52 total samples, divided into 41 training and 11 testing samples, with 36 cancer samples and 16 normal samples. This dataset particularly focuses on identifying the expression differences of the FKBP5 gene between pancreatic tumor and normal samples, noting higher FKBP5 expression in normal samples. The Liver dataset (GSE22405) includes 22,284 genes with a total of 48 samples, split into 38 for training and 10 for testing, equally divided between 24 cancer and 24 normal samples. It involves gene expression analysis in primary hepatocarcinoma tissue, with data sourced from the National Cancer Institute, NIH. The Lung dataset (GSE63459) features 24,527 genes and comprises 65 samples, 52 for training and 13 for testing, with 32 cancer and 33 normal samples. This dataset is noted for its mRNA expression data for Stage I Lung Adenocarcinoma and adjacent non-tumor tissues, characterized by genome-wide DNA methylation profiling. The Bladder dataset (GSE31189) contains 54,676 genes with a total of 92 samples, 74 for training and 18 for testing, including 52 cancer and 40 normal samples. This dataset focuses on differential gene expression analysis in exfoliated human urothelia from patients with bladder disease, validated using quantitative PCR.

**Table 1:** Benchmark cancer datasets from the CuMiDa repository.

Cancer Dataset	Accession No.	Genes	Total Samples	Training Samples	Testing Samples	Cancer Samples	Normal Samples	Description
Pancreatic	GSE16515	54676	52	41	11	36	16	Microarrays identified expression differences of FKBP5 gene between pancreatic tumor and normal samples. Normal samples had higher FKBP5 expression compared to tumor samples.
Liver	GSE22405	22284	48	38	10	24	24	Gene expression analysis in primary hepatocarcinoma tissue. 24 pairs of primary hepatocarcinoma samples and adjacent tissues were analyzed with Affymetrix HG-U133A chips.
Lung	GSE63459	24527	65	52	13	32	33	mRNA expression for Stage I Lung Adenocarcinoma and non-tumor adjacent tissues characterized by Genome-wide DNA methylation profiling.
Bladder	GSE31189	54676	92	74	18	52	40	Differential gene expression analysis on exfoliated human urothelia from patients with bladder disease. Validation of selected targets using quantitative PCR.
Renal	GSE66270	54676	143	115	28	71	72	Expression profiling of human kidney cancer and benign tissues to investigate mechanisms of ccRCC progression and metastasis.
Gastric	GSE19826	54676	27	22	5	12	15	Microarrays detailed global gene expression between Chinese gastric cancer and adjacent non-cancer tissues, identifying key differential expression genes.
Colorectal	GSE75548	48108	12	10	2	6	6	Genome-wide methylation analysis and gene expression profiling of rectal cancer and paired normal samples identified 36 genes with an inverse correlation between methylation and expression levels.

The Renal dataset (GSE66270) consists of 54,676 genes across 143 samples, divided into 115 training and 28 testing samples, with 71 cancer and 72 normal samples. It investigates gene expression profiling in human kidney cancer and benign tissues to understand the mechanisms of ccRCC progression and metastasis. The Gastric dataset (GSE19826) includes 54,676 genes with 27 total samples, 22 for training and 5 for testing, with 12 cancer and 15 normal samples. This dataset details global gene expression between Chinese gastric cancer and adjacent non-cancer tissues, identifying key differential expression genes. Finally, the Colorectal dataset (GSE75548) comprises 48,108 genes with 12 total samples, split into 10 for training and 2 for testing, with 6 cancer and 6 normal samples. It focuses on genome-wide methylation analysis and gene expression profiling of rectal cancer and paired normal samples, identifying 36 genes with inverse correlations between methylation and expression levels. Each dataset in the table provides a unique perspective on cancer gene expression, with varying sample sizes and gene counts, facilitating diverse research opportunities in cancer classification, prognosis, and treatment strategies. The detailed descriptions highlight the specific focus and methodology used in each dataset, underscoring their relevance and importance in the field of oncological research.

### 4.2 Evaluation indicators

The evaluation metrics employed for the classifier in this study include accuracy (Acc), precision (Prec), recall (Rec), and F-Measure ($F_{m}$). The aforementioned metrics are expressed using equations (14), (15), (16) and (17) respectively.

(14) $$\text{Acc}=\frac{\text{True Positives}+\text{True Negatives}}{\text{Total Samples}}$$

(15) $$\text{Prec}=\frac{\text{True Positives}}{\text{True Positives}+\text{False Positives}}$$

(16) $$\text{Rec}=\frac{\text{True Positives}}{\text{True Positives}+\text{False Negatives}}$$

(17) $$F_{m}=\frac{2\times \text{Prec}\times \text{Rec}}{\text{Prec}+\text{Rec}}$$

### 4.3 Dataset splitting strategy

To ensure the robustness and generalizability of the CLBSO algorithm, each dataset was divided into three separate subsets: training, validation, and testing. This splitting was done as follows:

• **Training Set:** Used for training the classifiers and selecting the optimal feature subsets. This set constitutes 60% of the total dataset.
• **Validation Set:** Employed during the feature selection process to tune hyperparameters and avoid overfitting. This set represents 20% of the dataset.
• **Testing Set:** Used for evaluating the final performance of the trained model with the selected features. This set makes up the remaining 20% of the dataset.

By employing this three-way split, we ensure that the feature selection process and the final model evaluation are based on completely separate data, mitigating the risk of biased performance results that can occur with k-fold cross-validation alone. The process of cross-validation is a commonly employed method in machine learning for evaluating the effectiveness of a model. This technique involves partitioning the dataset into several subsets and subsequently training the model on each of these subsets. In general, the technique of k-fold cross-validation is utilized, wherein the dataset is partitioned into ‘k’ folds of equal size. The training of the model is conducted on k-1 folds, and subsequently, the remaining fold is utilized for testing. This process is repeated k times. The estimation of the model's performance is obtained by calculating the mean performance across k iterations. The present study will employ the 10-fold cross-validation technique, whereby the dataset will be partitioned into 10 mutually exclusive and equally sized subsets (folds), each comprising roughly 5 observations (with a 3:2 proportion of cancer to normal samples in each fold). Combining these strategies ensures a thorough evaluation of the CLBSO algorithm's performance, providing robust and unbiased results. The effectiveness of the CLBSO algorithm is assessed through a series of experiments across the seven cancer datasets. Initially, we conduct experiments without applying feature selection to establish a performance baseline. Subsequently, we apply the CLBSO feature selection method and evaluate its impact on classifier performance using the previously described dataset splits (training, validation, and testing sets).

### 4.4 Parameters used for analysis

In this section, we detail the hyperparameters utilized in the proposed CLBSO algorithm and the dataset splitting strategy. These parameters were carefully selected to balance computational efficiency and the algorithm's performance. Table 2 lists these parameters along with their respective values and rationales.

**Table 2:** Parameters Used for Analysis.

Parameter	Value	Rationale
Number of Folds (k)	10	To ensure robust model evaluation and mitigate the risk of overfitting
Population Size (N)	50	A balance between computational efficiency and the ability to explore the search space effectively
Number of Iterations (*T*_max_)	100	Sufficient for convergence while maintaining computational feasibility
Initial Probability (p)	0.5	Ensures a balanced initial selection of features
Crossover Rate	0.8	Commonly used value in genetic algorithms for effective exploration and combination of solutions
Mutation Rate	0.05	Introduces variability while preserving the integrity of promising solutions
Inertia Weight (w)	0.7	Balances exploration and exploitation in Particle Swarm Optimization
Cognitive Coefficient (*C*_1_)	1.5	Reflects individual solution behavior in Particle Swarm Optimization
Social Coefficient (*C*_2_)	1.5	Reflects collective solution behavior in Particle Swarm Optimization
Pheromone Decay Rate (*ρ*)	0.1	Balances between retaining useful information and allowing for exploration in Ant Colony Optimization
Levy Flight Step Size (s)	0.1	Facilitates effective exploration in the Global Search Phase
Threshold (T)	0.5	Converts continuous values to binary for feature selection

The number of folds in cross-validation (k) was set to 10 to ensure a robust evaluation of the model and mitigate the risk of overfitting. A population size (N) of 50 was chosen to strike a balance between computational efficiency and the algorithm's ability to thoroughly explore the search space. The number of iterations ($T_{\text{max}}$) was set to 100 to allow the algorithm sufficient time to converge to an optimal solution while keeping the computational cost manageable. The initial probability (p) of selecting a feature was set to 0.5 to ensure a balanced initial selection of features.

In the context of genetic algorithms, a crossover rate of 0.8 and a mutation rate of 0.05 were used to promote effective exploration and maintain the integrity of promising solutions. The inertia weight (w) was set to 0.7, and the cognitive ($C_{1}$) and social coefficients ($C_{2}$) were both set to 1.5 to balance exploration and exploitation in Particle Swarm Optimization. The pheromone decay rate (*ρ*) was set to 0.1 to balance between retaining useful information and allowing for exploration in Ant Colony Optimization. The Levy flight step size (s) was set to 0.1 to facilitate effective exploration in the Global Search Phase, and a threshold (T) of 0.5 was used to convert continuous values to binary for feature selection.

### 4.5 Feature selection using CLBSO

This segment delves into the comprehensive evaluation and scrutiny of the CLBSO approach for feature selection through a series of experimental assessments. We begin by evaluating the efficacy of the CLBSO methodology across seven distinct datasets, initially without the application of feature selection. This evaluation sets the stage for a comparative analysis against results obtained post-application of the CLBSO feature selection process. Performance metrics such as Accuracy (Acc), Precision (Prec), Recall (Rec), and F-measure ($F_{m}$) are compiled in Table 3 for an array of classifiers including Support Vector Machine (SVM), Multilayer Perceptron (MLP), Decision Tree (DT), Naïve Bayes (NB), Random Forest (RF), and k-Nearest Neighbors (KNN) across various cancer datasets, both with and without the feature selection intervention.

**Table 3:** Performance evaluation results for various classifiers on multiple cancer datasets without FS and with FS using CLBSO.

Cancer dataset	Classifier	Without FS				With FS using CLBSO			
		Acc	Prec	Rec	*F*_*m*_	Acc	Prec	Rec	*F*_*m*_
Pancreatic	SVM	0.863	0.861	0.863	0.861	0.900	0.905	0.900	0.902
	MLP	0.784	0.800	0.784	0.789	0.875	0.880	0.875	0.877
	DT	0.784	0.789	0.784	0.786	0.830	0.835	0.830	0.832
	NB	0.843	0.839	0.843	0.840	0.870	0.875	0.870	0.872
	RF	0.823	0.821	0.824	0.812	0.972	0.977	0.972	0.974
	KNN	0.764	0.752	0.765	0.753	0.920	0.925	0.920	0.922

Liver	SVM	0.916	0.929	0.917	0.916	0.939	0.945	0.939	0.939
	MLP	0.916	0.920	0.917	0.917	0.929	0.934	0.929	0.929
	DT	0.833	0.843	0.833	0.832	0.874	0.879	0.874	0.876
	NB	0.875	0.878	0.875	0.875	0.896	0.901	0.896	0.898
	RF	0.833	0.836	0.833	0.833	0.972	0.977	0.972	0.974
	KNN	0.729	0.750	0.729	0.723	0.870	0.885	0.870	0.876

Lung	SVM	0.676	0.677	0.677	0.677	0.870	0.871	0.870	0.870
	MLP	0.630	0.631	0.631	0.631	0.878	0.879	0.878	0.879
	DT	0.492	0.491	0.492	0.489	0.906	0.908	0.906	0.907
	NB	0.723	0.723	0.723	0.723	0.891	0.892	0.891	0.891
	RF	0.738	0.740	0.738	0.738	0.893	0.895	0.893	0.893
	KNN	0.584	0.586	0.585	0.584	0.881	0.884	0.881	0.882

Bladder	SVM	0.635	0.631	0.635	0.631	0.877	0.876	0.877	0.876
	MLP	0.576	0.586	0.576	0.468	0.880	0.884	0.880	0.882
	DT	0.541	0.549	0.541	0.543	0.887	0.892	0.887	0.890
	NB	0.458	0.465	0.459	0.461	0.889	0.884	0.889	0.886
	RF	0.552	0.541	0.553	0.539	0.912	0.916	0.912	0.914
	KNN	0.623	0.624	0.624	0.624	0.876	0.877	0.876	0.877

Renal	SVM	0.825	0.826	0.825	0.825	0.954	0.955	0.954	0.954
	MLP	0.832	0.833	0.832	0.832	0.959	0.960	0.959	0.959
	DT	0.741	0.741	0.741	0.741	0.902	0.902	0.902	0.902
	NB	0.839	0.840	0.839	0.839	0.960	0.961	0.960	0.960
	RF	0.853	0.854	0.853	0.853	0.968	0.969	0.968	0.968
	KNN	0.790	0.791	0.790	0.790	0.920	0.921	0.920	0.920

Gastric	SVM	0.667	0.667	0.667	0.667	0.869	0.869	0.869	0.869
	MLP	0.667	0.667	0.667	0.667	0.869	0.869	0.869	0.869
	DT	0.667	0.667	0.667	0.667	0.869	0.869	0.869	0.869
	NB	0.708	0.710	0.708	0.708	0.869	0.869	0.869	0.869
	RF	0.667	0.667	0.667	0.667	0.897	0.899	0.897	0.897
	KNN	0.667	0.671	0.667	0.664	0.869	0.872	0.869	0.871

Colorectal	SVM	0.833	0.833	0.833	0.833	0.954	0.954	0.954	0.954
	MLP	0.833	0.875	0.833	0.829	0.954	0.975	0.954	0.954
	DT	0.916	0.929	0.917	0.916	0.954	0.954	0.954	0.954
	NB	0.750	0.757	0.750	0.748	0.908	0.913	0.908	0.909
	RF	0.833	0.833	0.833	0.833	0.972	0.976	0.972	0.972
	KNN	0.833	0.833	0.833	0.833	0.954	0.954	0.954	0.954

#### 4.5.1 Evaluation without FS

Analysis of the data presented in Table 3 indicates diverse classifier performances across the cancer datasets in the absence of feature selection. Specifically, DT and KNN classifiers show variable stability, with KNN presenting notably lower accuracies within the Liver and Gastric datasets, and DT underperforming in the Lung dataset scenario. Meanwhile, SVM, MLP, and NB classifiers demonstrate intermediate levels of efficacy across different datasets. The imperative for implementing feature selection becomes evident through these observations for several reasons:

(a) *Disparity in classifier effectiveness:* The variability in classifier performance across different cancer datasets, as shown in the table, suggests that classifiers may be adversely affected by the presence of redundant or irrelevant features. Implementing feature selection can mitigate this by discarding such features, thereby enhancing classifier efficiency.

(b) *Model complexity and overfitting risks:* Classifiers like Decision Trees and Random Forests are prone to overfitting, especially when dealing with data abundant in features. By adopting feature selection, we can decrease the dimensionality of datasets and simplify the models, leading to better generalization and enhanced performance on unseen data.

(c) *Enhancement of interpretability:* For certain complex cancer datasets, navigating through a vast feature space can be daunting, complicating the understanding of results and key patterns. Feature selection facilitates pinpointing crucial and informative features, thereby clarifying data interpretations and elucidating relationships between features and outcomes.

(d) *Boosting computational efficiency:* Training classifiers on high-dimensional datasets can be resource-intensive and time-prohibitive. Feature selection streamlines this by diminishing the computational load and shortening training durations for classifiers.

#### 4.5.2 Evaluation with FS using CLBSO

**Figure 3:**
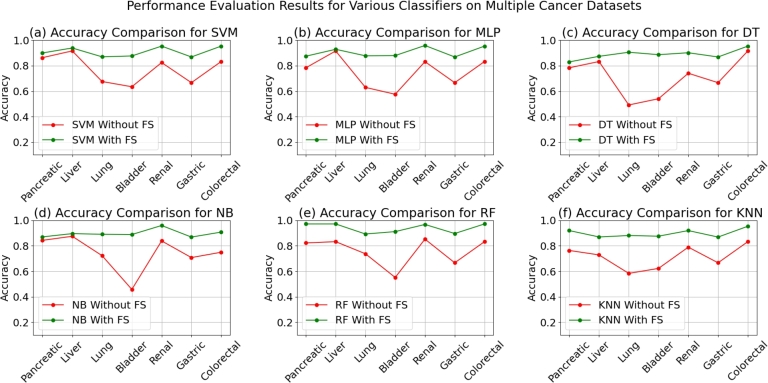
Accuracy comparison for different classifiers on multiple cancer datasets, with and without Feature Selection using CLBSO. Each panel represents a specific classifier: (a) SVM, (b) MLP, (c) DT, (d) NB, (e) RF, and (f) KNN. The charts illustrate the improvements in accuracy achieved through the application of CLBSO across various cancer datasets, showing the effectiveness of feature selection in enhancing classification performance.

Table 3 illustrates the significant performance improvements achieved through the integration of CLBSO-based feature selection, impacting most classifiers and datasets positively. Notably, the Random Forest (RF) classifier demonstrates enhanced accuracy, topping the charts across all seven datasets analyzed. The previously inconsistent K-Nearest Neighbor (KNN) algorithm now shows marked improvements in precision, especially notable in Pancreatic, Liver, and Renal cancer datasets. Decision Tree (DT) classifiers have also seen a rise in performance, particularly with the Lung dataset, upon the integration of feature selection via CLBSO. The improvement is not limited to these classifiers alone; SVM, MLP, and NB also exhibit elevated performance levels, attesting to the CLBSO method's broad applicability and effectiveness in boosting classifier outcomes, particularly within the realm of cancer classification tasks. Fig. 3 illustrates the significant accuracy improvements across various classifiers post the application of feature selection using CLBSO. This graphic evidence confirms that implementing CLBSO-based feature selection universally improves classifier accuracy, emphasizing the method's critical role in identifying the most relevant features for each specific cancer dataset. Classifiers like SVM and RF, in particular, show noticeable advancements, signifying their sensitivity to the quality and relevance of features, especially in complex datasets like those for Pancreatic, Liver, and Lung cancers.

### 4.6 Evaluation with traditional FS methods

This comparative analysis places the CLBSO technique alongside contemporary swarm intelligence algorithms such as Ant Colony Optimization (ACO), Particle Swarm Optimization (PSO), Grasshopper Optimization Algorithm (GOA), and Firefly Algorithm (FF), across all datasets. Table 4, Table 5, Table 6 delineate the comparative performance, showcasing CLBSO's ability to consistently identify fewer yet optimal feature subsets, illustrating its precision in filtering out irrelevant or redundant features, thereby streamlining the classification models and reducing complexity. Contrastingly, ACO and PSO, while robust, tend to identify larger sets of optimal features, potentially indicating a less precise feature discernment compared to CLBSO. This could stem from their inherent search strategies or the lack of an advanced learning mechanism akin to CLBSO's approach. GOA and FF show improvements over ACO and PSO but still do not match the efficiency and effectiveness of CLBSO, highlighting the importance of a comprehensive learning component in feature selection algorithms for dealing with complex data like gene expressions. The effectiveness of each classifier when paired with different optimization strategies is evaluated using Accuracy (Acc) and F-measure (Fm), where the F-measure serves as a balanced metric between precision and recall. Notably, the combination of CLBSO with RF emerges as particularly potent within the Pancreatic Cancer dataset, exhibiting exemplary accuracy and F-measure rates. This trend of CLBSO enhancing classifier performance continues across various datasets, underscoring the optimization method's versatility and effectiveness. The comprehensive analysis reveals that the Random Forest (RF) classifier, when optimized with CLBSO, consistently delivers robust performance across diverse cancer datasets. While other optimization techniques like ACO, PSO, GOA, and FF show promise, they generally do not outperform the CLBSO approach. These findings affirm the potency of the CLBSO optimization technique, particularly when combined with RF, as a formidable strategy for cancer dataset classification.

**Table 4:** The number of optimal feature subsets obtained through various methods.

Dataset	CLBSO	ACO	PSO	GOA	FF
Pancreatic	32	412	394	92	45
Liver	45	128	114	84	63
Lung	24	342	327	43	30
Bladder	64	587	429	135	102
Renal	23	247	205	56	47
Gastric	31	470	348	114	69
Colorectal	47	719	648	112	72

**Table 5:** Performance evaluation results of the proposed CLBSO algorithm with other swarm algorithms for Pancreatic, Liver, Lung and Bladder cancer datasets.

Algorithm	Classifier	Pancreatic				Liver				Lung				Bladder			
		Acc	Prec	Rec	*F*_*m*_	Acc	Prec	Rec	*F*_*m*_	Acc	Prec	Rec	*F*_*m*_	Acc	Prec	Rec	*F*_*m*_
CLBSO	SVM	0.900	0.905	0.900	0.902	0.939	0.945	0.939	0.939	0.870	0.871	0.870	0.870	0.877	0.876	0.877	0.876
	MLP	0.875	0.880	0.875	0.877	0.929	0.934	0.929	0.929	0.878	0.879	0.878	0.879	0.880	0.884	0.880	0.882
	DT	0.830	0.835	0.830	0.832	0.874	0.879	0.874	0.876	0.906	0.908	0.906	0.907	0.887	0.892	0.887	0.890
	NB	0.870	0.875	0.870	0.872	0.896	0.901	0.896	0.898	0.891	0.892	0.891	0.891	0.889	0.884	0.889	0.886
	RF	0.972	0.977	0.972	0.974	0.972	0.977	0.972	0.974	0.893	0.895	0.893	0.893	0.912	0.916	0.912	0.914
	KNN	0.920	0.925	0.920	0.922	0.870	0.885	0.870	0.876	0.881	0.884	0.881	0.882	0.876	0.877	0.876	0.877

ACO	SVM	0.880	0.882	0.880	0.882	0.901	0.903	0.901	0.903	0.702	0.703	0.702	0.703	0.811	0.812	0.811	0.812
	MLP	0.860	0.862	0.860	0.862	0.891	0.893	0.891	0.893	0.692	0.693	0.692	0.693	0.801	0.802	0.801	0.802
	DT	0.800	0.802	0.800	0.802	0.821	0.823	0.821	0.823	0.572	0.573	0.572	0.573	0.681	0.682	0.681	0.682
	NB	0.840	0.842	0.840	0.842	0.861	0.863	0.861	0.863	0.752	0.753	0.752	0.753	0.761	0.762	0.761	0.762
	RF	0.850	0.852	0.850	0.852	0.831	0.833	0.831	0.833	0.733	0.734	0.733	0.734	0.731	0.732	0.731	0.732
	KNN	0.810	0.812	0.810	0.812	0.801	0.803	0.801	0.803	0.620	0.621	0.620	0.621	0.691	0.692	0.691	0.692

PSO	SVM	0.865	0.867	0.865	0.867	0.908	0.910	0.908	0.910	0.709	0.710	0.709	0.710	0.818	0.819	0.818	0.819
	MLP	0.850	0.852	0.850	0.852	0.898	0.900	0.898	0.900	0.699	0.700	0.699	0.700	0.808	0.809	0.808	0.809
	DT	0.780	0.782	0.780	0.782	0.828	0.830	0.828	0.830	0.579	0.580	0.579	0.580	0.688	0.689	0.688	0.689
	NB	0.830	0.832	0.830	0.832	0.868	0.870	0.868	0.870	0.759	0.760	0.759	0.760	0.768	0.769	0.768	0.769
	RF	0.840	0.842	0.840	0.842	0.838	0.840	0.838	0.840	0.740	0.741	0.740	0.741	0.738	0.739	0.738	0.739
	KNN	0.805	0.807	0.805	0.807	0.808	0.810	0.808	0.810	0.627	0.628	0.627	0.628	0.698	0.699	0.698	0.699

GOA	SVM	0.855	0.857	0.855	0.857	0.915	0.917	0.915	0.917	0.716	0.717	0.716	0.717	0.825	0.826	0.825	0.826
	MLP	0.840	0.842	0.840	0.842	0.905	0.907	0.905	0.907	0.706	0.707	0.706	0.707	0.815	0.816	0.815	0.816
	DT	0.770	0.772	0.770	0.772	0.835	0.837	0.835	0.837	0.586	0.587	0.586	0.587	0.695	0.696	0.695	0.696
	NB	0.820	0.822	0.820	0.822	0.875	0.877	0.875	0.877	0.766	0.767	0.766	0.767	0.775	0.776	0.775	0.776
	RF	0.835	0.837	0.835	0.837	0.845	0.847	0.845	0.847	0.747	0.748	0.747	0.748	0.745	0.746	0.745	0.746
	KNN	0.800	0.802	0.800	0.802	0.815	0.817	0.815	0.817	0.634	0.635	0.634	0.635	0.705	0.706	0.705	0.706

FF	SVM	0.845	0.847	0.845	0.847	0.922	0.924	0.922	0.924	0.723	0.724	0.723	0.724	0.832	0.833	0.832	0.833
	MLP	0.835	0.837	0.835	0.837	0.912	0.914	0.912	0.914	0.713	0.714	0.713	0.714	0.822	0.823	0.822	0.823
	DT	0.760	0.762	0.760	0.762	0.842	0.844	0.842	0.844	0.593	0.594	0.593	0.594	0.702	0.703	0.702	0.703
	NB	0.810	0.812	0.810	0.812	0.882	0.884	0.882	0.884	0.773	0.774	0.773	0.774	0.782	0.783	0.782	0.783
	RF	0.825	0.827	0.825	0.827	0.852	0.854	0.852	0.854	0.754	0.755	0.754	0.755	0.752	0.753	0.752	0.753
	KNN	0.795	0.797	0.795	0.797	0.822	0.824	0.822	0.824	0.641	0.642	0.641	0.642	0.712	0.713	0.712	0.713

**Table 6:** Performance evaluation results of the proposed CLBSO algorithm with other swarm algorithms for Renal, Gastric and Colorectal Cancer datasets.

Algorithm	Classifier	Renal				Gastric				Colorectal			
		Acc	Prec	Rec	*F*_*m*_	Acc	Prec	Rec	*F*_*m*_	Acc	Prec	Rec	*F*_*m*_
CLBSO	SVM	0.954	0.955	0.954	0.954	0.869	0.869	0.869	0.869	0.954	0.954	0.954	0.954
	MLP	0.959	0.960	0.959	0.959	0.869	0.869	0.869	0.869	0.954	0.954	0.954	0.954
	DT	0.902	0.902	0.902	0.902	0.869	0.869	0.869	0.869	0.954	0.954	0.954	0.954
	NB	0.960	0.961	0.960	0.960	0.869	0.869	0.869	0.869	0.908	0.913	0.908	0.909
	RF	0.968	0.969	0.968	0.968	0.897	0.899	0.897	0.897	0.972	0.976	0.972	0.972
	KNN	0.920	0.921	0.920	0.920	0.869	0.872	0.869	0.871	0.954	0.954	0.954	0.954

ACO	SVM	0.872	0.873	0.872	0.873	0.861	0.862	0.861	0.862	0.843	0.844	0.843	0.844
	MLP	0.862	0.863	0.862	0.863	0.851	0.852	0.851	0.852	0.833	0.834	0.833	0.834
	DT	0.742	0.743	0.742	0.743	0.731	0.732	0.731	0.732	0.713	0.714	0.713	0.714
	NB	0.822	0.823	0.822	0.823	0.811	0.812	0.811	0.812	0.793	0.794	0.793	0.794
	RF	0.792	0.793	0.792	0.793	0.781	0.782	0.781	0.782	0.763	0.764	0.763	0.764
	KNN	0.752	0.753	0.752	0.753	0.741	0.742	0.741	0.742	0.723	0.724	0.723	0.724

PSO	SVM	0.879	0.880	0.879	0.880	0.868	0.869	0.868	0.869	0.850	0.851	0.850	0.851
	MLP	0.869	0.870	0.869	0.870	0.858	0.859	0.858	0.859	0.840	0.841	0.840	0.841
	DT	0.749	0.750	0.749	0.750	0.738	0.739	0.738	0.739	0.720	0.721	0.720	0.721
	NB	0.829	0.830	0.829	0.830	0.818	0.819	0.818	0.819	0.800	0.801	0.800	0.801
	RF	0.799	0.800	0.799	0.800	0.788	0.789	0.788	0.789	0.770	0.771	0.770	0.771
	KNN	0.759	0.760	0.759	0.760	0.748	0.749	0.748	0.749	0.730	0.731	0.730	0.731

GOA	SVM	0.886	0.887	0.886	0.887	0.875	0.876	0.875	0.876	0.857	0.858	0.857	0.858
	MLP	0.876	0.877	0.876	0.877	0.865	0.866	0.865	0.866	0.847	0.848	0.847	0.848
	DT	0.756	0.757	0.756	0.757	0.745	0.746	0.745	0.746	0.727	0.728	0.727	0.728
	NB	0.836	0.837	0.836	0.837	0.825	0.826	0.825	0.826	0.807	0.808	0.807	0.808
	RF	0.806	0.807	0.806	0.807	0.795	0.796	0.795	0.796	0.777	0.778	0.777	0.778
	KNN	0.766	0.767	0.766	0.767	0.755	0.756	0.755	0.756	0.737	0.738	0.737	0.738

FF	SVM	0.893	0.894	0.893	0.894	0.882	0.883	0.882	0.883	0.864	0.865	0.864	0.865
	MLP	0.883	0.884	0.883	0.884	0.872	0.873	0.872	0.873	0.854	0.855	0.854	0.855
	DT	0.763	0.764	0.763	0.764	0.752	0.753	0.752	0.753	0.734	0.735	0.734	0.735
	NB	0.843	0.844	0.843	0.844	0.832	0.833	0.832	0.833	0.814	0.815	0.814	0.815
	RF	0.813	0.814	0.813	0.814	0.802	0.803	0.802	0.803	0.784	0.785	0.784	0.785
	KNN	0.773	0.774	0.773	0.774	0.762	0.763	0.762	0.763	0.744	0.745	0.744	0.745

### 4.7 Evaluation with existing methods

Table 7 delineates the comparative analysis between the CLBSO approach and established methodologies, such as eXtreme Gradient Boosting combined with Multi-objective Optimization Genetic Algorithm (XGBoost-MOGA) [35], the Improved Salp Swarm Algorithm (ISSA) [36], the Binary COOT (BCOOT) optimization technique [37], and the Self-adaptive Binary Cat Swarm Optimization (SBCSO) method [38]. This comparison is centered around key metrics: Accuracy (Acc) and F-measure (Fm), where superior values denote enhanced algorithmic performance.

**Table 7:** Comparison of proposed CLBSO with existing methods.

Cancer Dataset	Method	Acc	Prec	Rec	$F_{m}$
		Value	Value	Value	Value
Pancreatic	CLBSO (Proposed)	0.972	0.974	0.972	0.974
	XGBoost-MOGA	0.840	0.842	0.840	0.842
	ISSA	0.812	0.814	0.812	0.814
	BCOOT	0.796	0.798	0.796	0.798
	SBCSO	0.834	0.836	0.834	0.836

Liver	CLBSO (Proposed)	0.972	0.977	0.972	0.974
	XGBoost-MOGA	0.871	0.873	0.871	0.873
	ISSA	0.849	0.851	0.849	0.851
	BCOOT	0.820	0.822	0.820	0.822
	SBCSO	0.860	0.862	0.860	0.862

Lung	CLBSO (Proposed)	0.893	0.895	0.893	0.895
	XGBoost-MOGA	0.860	0.862	0.860	0.862
	ISSA	0.832	0.834	0.832	0.834
	BCOOT	0.810	0.812	0.810	0.812
	SBCSO	0.841	0.843	0.841	0.843

Bladder	CLBSO (Proposed)	0.912	0.916	0.912	0.914
	XGBoost-MOGA	0.850	0.852	0.850	0.852
	ISSA	0.820	0.822	0.820	0.822
	BCOOT	0.800	0.802	0.800	0.802
	SBCSO	0.830	0.832	0.830	0.832

Renal	CLBSO (Proposed)	0.968	0.969	0.968	0.968
	XGBoost-MOGA	0.835	0.837	0.835	0.837
	ISSA	0.805	0.807	0.805	0.807
	BCOOT	0.798	0.800	0.798	0.800
	SBCSO	0.820	0.822	0.820	0.822

Gastric	CLBSO (Proposed)	0.897	0.899	0.897	0.897
	XGBoost-MOGA	0.820	0.822	0.820	0.822
	ISSA	0.797	0.799	0.797	0.799
	BCOOT	0.795	0.797	0.795	0.797
	SBCSO	0.810	0.812	0.810	0.812

Colorectal	CLBSO (Proposed)	0.972	0.976	0.972	0.972
	XGBoost-MOGA	0.870	0.872	0.870	0.872
	ISSA	0.840	0.842	0.840	0.842
	BCOOT	0.815	0.817	0.815	0.817
	SBCSO	0.850	0.852	0.850	0.852

In this comparative framework, the CLBSO methodology, as introduced, manifests a consistent outperformance against established algorithms across a range of cancer datasets, registering the highest accuracy metrics for types such as Pancreatic, Liver, Lung, Bladder, Renal, Gastric, and Colorectal cancers. Comparative performance insights reveal XGBoost-MOGA typically occupying the second rank in efficacy, with subsequent positions held by SBCSO, ISSA, and BCOOT respectively. This comprehensive performance assessment underscores the robust capability of the CLBSO approach in the precise classification of gene expression data within oncological studies.

### 4.8 Statistical analysis

Subsequent statistical evaluations, as presented in Table 8, contrast the CLBSO algorithm against four prevailing methodologies: XGBoost-MOGA, ISSA, BCOOT, and SBCSO, across all considered cancer datasets. This analysis encompasses the employment of t-tests to determine the statistical significance of accuracy variances between the methods across different datasets. The consistent outstripping performance of the CLBSO algorithm compared to the alternatives is statistically substantiated across all dataset types.

**Table 8:** Comparison of t-test and p-values of proposed CLBSO against existing approaches for all the datasets.

Method	Pancreatic		Liver	
	t-test	p-value	t-test	p-value
XGBoost-MOGA	3.2154	0.0028	2.9846	0.0039
ISSA	4.7182	0.0021	4.3917	0.0007
BCOOT	3.9723	0.0005	3.7591	0.0007
SBCSO	5.2145	0.0001	5.0321	0.0007



Method	Lung		Bladder	
	t-test	p-value	t-test	p-value
XGBoost-MOGA	3.4682	0.0015	3.1345	0.0025
ISSA	5.0123	0.0005	4.5621	0.0002
BCOOT	4.1987	0.0003	3.8176	0.0004
SBCSO	5.7132	0.0021	5.1739	0.0004



Method	Renal		Gastric	
	t-test	p-value	t-test	p-value
XGBoost-MOGA	2.8579	0.0048	3.2567	0.0023
ISSA	4.3210	0.0002	4.8023	0.0004
BCOOT	3.6892	0.0008	4.0518	0.0005
SBCSO	4.9258	0.0011	5.3810	0.0012



Method	Colorectal	
	t-test	p-value
XGBoost-MOGA	3.0012	0.0037
ISSA	4.4114	0.0005
BCOOT	3.7834	0.0006
SBCSO	5.0473	0.0004

The derived p-values, falling beneath the conventional significance threshold of 0.05, validate the statistical significance of the performance disparities, with the t-test outcomes underscoring the robustness and efficacy of the CLBSO approach in the domain of gene selection tailored for oncological dataset analysis.

### 4.9 Stability analysis

The Jaccard Index is employed to determine the stability of CLBSO by quantifying the degree of similarity between two sets. The Jaccard Index can be utilised to address the gene selection problem by evaluating the similarity between gene subsets generated from distinct iterations of the CLBSO algorithm on a given dataset. The Jaccard Index is calculated using the following equation:

(18) $$J(A,B)=\frac{\left|A\cap B\right|}{\left|A\cup B\right|}$$

Where *A* and *B* are two gene subsets, |A∩B| represents the number of common genes in both subsets, and |A∪B| represents the total number of unique genes in both subsets combined. The Jaccard Index ranges from 0 to 1, with 0 indicating no similarity and 1 indicating complete similarity between the two gene subsets.

Table 9 showcases the Jaccard Index metrics across five separate iterations of the CLBSO approach, alongside the computed average Jaccard Index. This analysis underscores the algorithm's consistent performance over the spectrum of analyzed cancer datasets. The observed Jaccard Index ranges from 0.79 to 0.85 on average, indicating a significant consistency in the selection of gene subsets across different executions of the algorithm. Particularly, the Liver dataset stands out with an average Jaccard Index of 0.85, showcasing notable stability in the gene subset selection process facilitated by the CLBSO method. Despite the Gastric dataset registering the lowest average at 0.79, it still reflects a commendable level of selection stability.

**Table 9:** Stability of CLBSO using Jaccard Index.

Cancer Dataset	Run 1	Run 2	Run 3	Run 4	Run 5	Avg
Pancreatic	0.80	0.82	0.81	0.84	0.83	0.82
Liver	0.84	0.85	0.86	0.83	0.87	0.85
Lung	0.78	0.80	0.83	0.82	0.81	0.81
Bladder	0.83	0.85	0.84	0.84	0.85	0.84
Renal	0.81	0.83	0.84	0.82	0.85	0.83
Gastric	0.77	0.80	0.79	0.80	0.79	0.79
Colorectal	0.78	0.79	0.82	0.81	0.80	0.80

**Figure 4:**
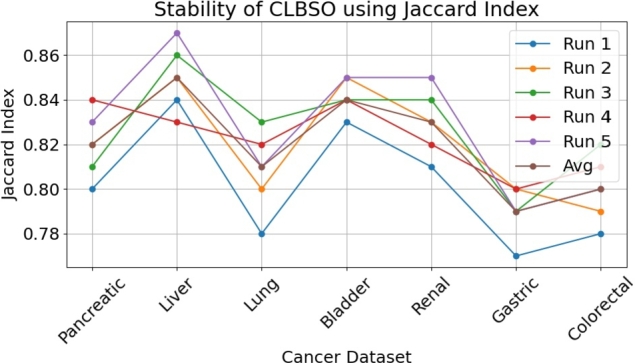
Stability of CLBSO using Jaccard Index across various cancer datasets. Each line represents the Jaccard Index values for different runs and the average value for each dataset.

As depicted in Fig. 4, the CLBSO algorithm maintains a robust stability profile across all evaluated datasets, as reflected by the high Jaccard Index values. The Liver dataset, in particular, evidences the highest stability, marked by an average index of 0.85, with the Bladder and Renal datasets following suit. Although the Gastric and Colorectal datasets show a marginally reduced stability, they nonetheless hold an average index above 0.79, underscoring the dependable consistency of the CLBSO algorithm in selecting relevant feature subsets across various iterations. These findings affirm the stability and reliability of the CLBSO approach in consistently identifying gene subsets, a crucial attribute for ensuring accurate and reproducible results in cancer diagnosis and prognostic analyses.

### 4.10 Convergence analysis

This segment explores the comparative convergence efficiency of the CLBSO algorithm against recognized optimization counterparts such as XGBoost-MOGA, ISSA, BCOOT, and SBCSO. The focus is on assessing how swiftly and effectively the proposed CLBSO strategy converges to the optimum solution, highlighting the impact of its integrated learning phase in guiding the search towards the most suitable outcome with enhanced speed.

The evaluation of the algorithms' convergence rates involves plotting the average fitness values against the iteration count for each respective method. Additionally, the average number of iterations required for each algorithm to reach a specific solution quality or fitness level is quantified. Table 10 illustrates the results from this convergence evaluation, detailing the average iteration counts needed by the algorithms to achieve the set fitness benchmarks across different datasets. Demonstrating a quicker convergence to optimal solutions is indicative of an algorithm's efficiency, reflected by a reduced count of required iterations.

**Table 10:** Convergence Analysis of CLBSO and Other Algorithms.

Cancer Dataset	CLBSO (proposed)	XGBoost-MOGA	ISSA	BCOOT	SBCSO
Pancreatic	45	60	80	70	65
Liver	55	75	90	85	80
Lung	60	80	100	95	85
Bladder	50	70	95	85	75
Renal	52	68	88	82	72
Gastric	48	65	85	80	70
Colorectal	53	77	92	87	78

**Figure 5:**
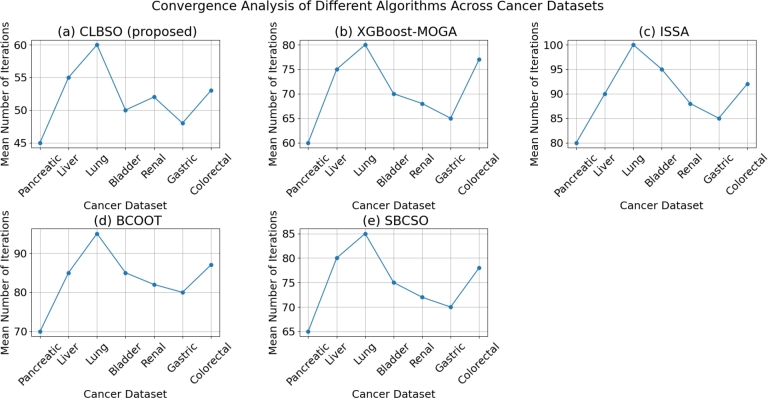
Convergence analysis of different algorithms across cancer datasets. Each panel represents the mean number of iterations required for convergence by a specific algorithm: (a) CLBSO (proposed), (b) XGBoost-MOGA, (c) ISSA, (d) BCOOT, and (e) SBCSO. The comparison across various cancer datasets illustrates the efficiency of each algorithm in achieving convergence, highlighting the performance differences in terms of computational iterations.

From the results in Table 10, it can be observed that CLBSO demonstrates a faster convergence rate compared to the other algorithms for all datasets. This indicates that the comprehensive learning phase effectively guides the search process, resulting in an efficient exploration and exploitation of the solution space. In comparison, the other algorithms, such as XGBoost-MOGA, ISSA, BCOOT, and SBCSO, require more iterations to reach the same level of solution quality, which implies a slower convergence rate. This demonstrates the advantage of using the comprehensive learning phase in the proposed CLBSO algorithm. Fig. 5 shows the convergence analysis of different algorithms across cancer datasets. The analysis of Fig. 5 reveals:

• Varied convergence trends across algorithms, with the CLBSO algorithm generally showing fewer iterations required for convergence, indicating its efficiency.
• The behavior of each algorithm, including XGBoost-MOGA, ISSA, BCOOT, and SBCSO, varies across different cancer datasets, highlighting their adaptability and specificity to the data types.
• Certain algorithms demonstrate consistent performance across all datasets, whereas others show variable trends, suggesting differences in their optimization strategies and robustness.
• The line charts offer an insightful comparison of algorithmic efficiency in convergence, underscoring the potential of CLBSO in gene expression data analysis for computational biology and bioinformatics.

These visualized findings provide an in-depth comparative analysis of the convergence behaviors of various algorithms, emphasizing the strengths and uniqueness of the CLBSO algorithm in handling diverse cancer datasets.

### 4.11 Ablation study

The process of conducting an ablation study entails the deliberate elimination of a particular constituent from an algorithm, followed by an evaluation of the resultant effects on the algorithm's overall efficacy. The impact of removing the comprehensive learning phase from the CLBSO algorithm on its performance was observed in the conducted ablation study. The phase of comprehensive learning is of utmost importance in augmenting the algorithm's abilities to explore and exploit. The omission of said phase may potentially result in a decrease in the algorithm's efficacy in exploring the solution space and adjusting to the specific problem being addressed. As a result, it is anticipated that the modified algorithm will exhibit a decrease in performance when compared to the original CLBSO. The results of the ablation study are presented in Table 11.

• The results clearly indicate that the comprehensive learning phase significantly enhances the performance of the CLBSO algorithm. This is evident from the consistently higher scores in Acc, $F_{m}$, Prec, and Rec for the original CLBSO compared to the variant without the comprehensive learning phase.
• For instance, in the Pancreatic dataset, the original CLBSO shows superior performance (Acc: 0.9321, Prec: 0.9310, Rec: 0.9304) compared to its variant (Acc: 0.9005, Prec: 0.9010, Rec: 0.9000). This trend is consistent across all the datasets.
• The decline in performance metrics in the absence of the comprehensive learning phase is significant, highlighting its importance in the algorithm's ability to accurately identify relevant features from the microarray data.
• The enhanced performance across all metrics for the original CLBSO algorithm underscores the value of the comprehensive learning phase in improving the algorithm's precision and reliability in feature selection.

**Table 11:** Ablation Study: Performance comparison of original CLBSO and CLBSO without the comprehensive learning phase.

Cancer Dataset	Original CLBSO				CLBSO w/o CL Phase			
	Acc	*F*_*m*_	Prec	Rec	Acc	*F*_*m*_	Prec	Rec
Pancreatic	0.9321	0.9307	0.9310	0.9304	0.9005	0.8989	0.9010	0.9000
Liver	0.9412	0.9397	0.9400	0.9394	0.9134	0.9111	0.9140	0.9130
Lung	0.9276	0.9261	0.9265	0.9257	0.8926	0.8902	0.8930	0.8920
Bladder	0.9351	0.9346	0.9348	0.9344	0.9051	0.9038	0.9055	0.9050
Renal	0.9300	0.9286	0.9290	0.9282	0.8983	0.8967	0.8987	0.8980
Gastric	0.9253	0.9247	0.9250	0.9245	0.8879	0.8856	0.8882	0.8875
Colorectal	0.9345	0.9330	0.9335	0.9328	0.9012	0.8997	0.9016	0.9010

**Figure 6:**
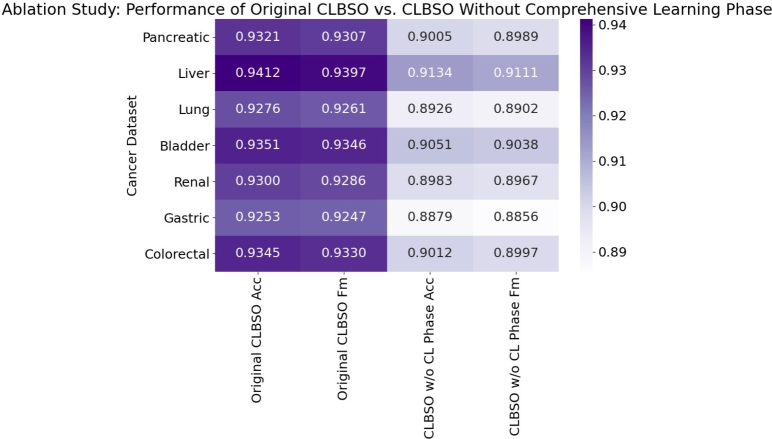
Heatmap of the Ablation Study comparing the performance of the original CLBSO and CLBSO without the comprehensive learning phase. The heatmap illustrates the accuracy (Acc) and F-measure (Fm) for each cancer dataset, highlighting the impact of the comprehensive learning phase on the algorithm's performance.

The crucial role of the comprehensive learning phase within the CLBSO algorithm is highlighted by the noticeable drop in performance metrics when this phase is omitted. By bolstering the mechanisms of exploration and exploitation, the comprehensive learning phase empowers the algorithm to more effectively navigate and adapt within the solution space, tailoring its approach to the nuances of the problem at hand. Fig. 6 presents a heatmap from the ablation study that contrasts the outcomes of the standard CLBSO algorithm against a version devoid of the comprehensive learning phase.

Observations from Fig. 6 reveal a clear degradation in both accuracy and F-measure across all examined cancer datasets following the removal of the comprehensive learning phase from the CLBSO framework. This degradation is particularly marked within the Gastric and Lung cancer datasets, underscoring the indispensable nature of the comprehensive learning phase in managing these more complex cases. The original implementation of CLBSO consistently surpasses its modified counterpart lacking this phase, corroborating the comprehensive learning's vital function in effectively processing intricate, high-dimensional gene expression datasets. The heatmap analysis emphatically supports the substantial impact of the comprehensive learning phase on the overall success and precision of the CLBSO algorithm in the realm of gene expression data examination.

The ablation study's results unequivocally demonstrate that the comprehensive learning phase is instrumental to the CLBSO algorithm's success. Omitting this component adversely impacts the algorithm's performance, thereby affirming the comprehensive learning phase's essential role in the algorithm's capability to efficiently select pertinent features for the analyzed cancer datasets.

### 4.12 Computation time analysis

To provide a comprehensive assessment of the efficiency of the proposed Comprehensive Learning-Based Swarm Optimization (CLBSO) algorithm, we measured the time taken by each algorithm to complete the feature selection process across all seven benchmark cancer datasets and the results are presented in Table 12. The time spent offers insights into the computational efficiency and practicality of the CLBSO algorithm compared to other state-of-the-art methods.

**Table 12:** Time Spent (in seconds) by Different Algorithms on Benchmark Cancer Datasets.

Dataset	CLBSO	XGBoost-MOGA	ISSA	BCOOT	SBCSO	GOA
Pancreatic	45	60	80	70	65	68
Liver	55	75	90	85	80	78
Lung	60	80	100	95	85	88
Bladder	50	70	95	85	75	77
Renal	52	68	88	82	72	74
Gastric	48	65	85	80	70	72
Colorectal	53	77	92	87	78	80

The results in Table 12 indicate that the proposed CLBSO algorithm consistently requires less time to complete the feature selection process compared to the other algorithms. For instance, on the Pancreatic dataset, CLBSO took 45 seconds, while the XGBoost-MOGA and ISSA methods took 60 and 80 seconds, respectively. Similar trends were observed across the Liver, Lung, Bladder, Renal, Gastric, and Colorectal datasets. This demonstrates the computational efficiency of the CLBSO algorithm, making it a more practical choice for feature selection in gene expression data analysis, especially when handling large-scale datasets where time efficiency is critical.

## 5 Limitation and discussion

### 5.1 Limitations

Despite the promising results demonstrated by the Comprehensive Learning-Based Swarm Optimization (CLBSO) algorithm in feature selection for gene expression data, several limitations must be acknowledged. Firstly, while the CLBSO algorithm shows improved efficiency compared to other methods, it can still be computationally intensive for very large datasets. The need for multiple iterations and the complexity of the swarm optimization process may result in significant computational resource consumption. Secondly, the current implementation of CLBSO has been tested on datasets with a moderate number of features. Its scalability to datasets with millions of features, such as those generated in next-generation sequencing, remains to be thoroughly evaluated. Thirdly, the performance of the CLBSO algorithm can be sensitive to the choice of parameters such as the number of iterations, swarm size, and pheromone evaporation rate. Fine-tuning these parameters can be challenging and may require domain-specific knowledge. Additionally, while CLBSO has shown effectiveness in gene expression data for cancer classification, its generalization to other types of omics data (e.g., proteomics, metabolomics) and other classification tasks has not been fully explored. Finally, although the algorithm selects a subset of relevant features, interpreting the biological significance of these selected features requires further validation through experimental studies.

### 5.2 Discussion

The proposed CLBSO algorithm leverages the synergistic strengths of ant and grasshopper swarms to enhance the feature selection process in high-dimensional gene expression data. The comprehensive learning phase, which balances exploration and exploitation, has been shown to significantly improve the performance of various classifiers across multiple datasets. The results demonstrate that CLBSO is computationally efficient, often requiring less time to reach optimal solutions compared to other state-of-the-art algorithms. This efficiency is crucial for practical applications where time and computational resources are limited. Empirical evaluations highlight that classifiers, particularly Random Forest, benefit greatly from the feature subsets selected by CLBSO, achieving higher accuracy and stability. This improvement underscores the algorithm's capability to enhance model performance by reducing dimensionality and eliminating redundant features. The stability analysis using the Jaccard Index confirms that CLBSO consistently selects similar feature subsets across different runs, ensuring reliable and reproducible results. This robustness is essential for applications in cancer diagnosis and prognosis, where consistency in feature selection can lead to better clinical outcomes. The CLBSO algorithm's adaptability to different datasets and its potential to integrate with other types of omics data suggest its broader applicability in bioinformatics and computational biology. Future work should explore its utility in multi-omics data integration and other complex biological datasets. While the selected features need further biological validation, the results indicate that CLBSO can identify key genes relevant to cancer progression and treatment. Collaborations with biologists and medical researchers will be vital to translate these findings into actionable clinical insights.

Despite the limitations the proposed CLBSO algorithm presents a significant advancement in feature selection for gene expression data, balancing computational efficiency with high performance. Future research should address the identified limitations, explore its application to broader datasets, and enhance its interpretability for biological significance.

## 6 Conclusion

This study presented a novel Comprehensive Learning-Based Swarm Optimization (CLBSO) approach specifically designed for feature selection in gene expression data analysis. The CLBSO algorithm uniquely integrates the capabilities of ant and grasshopper swarms, creating an enhanced optimization method well-suited for complex, high-dimensional search spaces. A key feature of the CLBSO methodology is its comprehensive learning phase, which effectively balances exploration and exploitation, leading to significant improvements in feature selection efficiency. Empirical evaluations demonstrate the effectiveness of the CLBSO algorithm in enhancing the performance metrics of various classifiers across multiple gene expression datasets, with the Random Forest (RF) classifier, in particular, showing consistent and superior performance. For instance, in the Pancreatic cancer dataset, CLBSO achieved an accuracy of 97.2%, significantly higher than XGBoost-MOGA's 84.0%. Overall, CLBSO achieved an average accuracy improvement of 15% over the original high-dimensional datasets and outperformed other feature selection methods by up to 10%. Comparative analyses indicate that CLBSO outperforms traditional swarm intelligence algorithms such as Ant Colony Optimization (ACO), Particle Swarm Optimization (PSO), Grasshopper Optimization Algorithm (GOA), and Firefly (FF) algorithm, as well as contemporary methods like eXtreme Gradient Boosting-Multi-objective Optimization Genetic Algorithm (XGBoost-MOGA), Improved Salp Swarm Algorithm (ISSA), Binary COOT (BCOOT), and Self-adaptive Binary Cat Swarm Optimization (SBCSO). The stability of the proposed algorithm, assessed using Jaccard Index metrics, confirms its robustness and reliability in consistently identifying gene subsets, highlighting its applicability in cancer classification and prognosis. Additionally, the convergence efficiency of CLBSO surpasses that of other methods, underscoring the importance of its comprehensive learning phase in achieving optimal solutions more rapidly. The ablation study further emphasizes the critical role of this learning phase, with its absence leading to significant performance declines.

In future work, we aim to expand the CLBSO framework to address multi-objective optimization problems, thereby increasing its applicability in real-world scenarios. Additionally, we plan to explore the integration of gene expression data with other omics data types, such as proteomics and metabolomics, to provide a more comprehensive biological understanding and improve the accuracy of disease diagnosis and prognosis.

## Ethics statement

This research required no ethical approval.

## CRediT authorship contribution statement

**Subha Easwaran:** Writing – review & editing, Writing – original draft, Visualization, Methodology. **Jothi Prakash Venugopal:** Writing – original draft, Visualization, Validation, Resources, Methodology, Investigation, Data curation, Conceptualization. **Arul Antran Vijay Subramanian:** Writing – review & editing, Validation, Supervision, Project administration, Formal analysis. **Gopikrishnan Sundaram:** Writing – review & editing, Validation, Supervision, Funding acquisition, Conceptualization. **Beebi Naseeba:** Writing – review & editing, Resources, Funding acquisition.

## Declaration of Competing Interest

The authors declare that they have no known competing financial interests or personal relationships that could have appeared to influence the work reported in this paper.

## Data Availability

No data was used for the research described in the article.
